# Different Impact of Definitions of Sarcopenia in Defining Frailty Status in a Population of Older Women with Early Breast Cancer

**DOI:** 10.3390/jpm11040243

**Published:** 2021-03-26

**Authors:** Andrea Bellieni, Domenico Fusco, Alejandro Martin Sanchez, Gianluca Franceschini, Beatrice Di Capua, Elena Allocca, Enrico Di Stasio, Fabio Marazzi, Luca Tagliaferri, Riccardo Masetti, Roberto Bernabei, Giuseppe Ferdinando Colloca

**Affiliations:** 1 Dipartimento di Scienze dell’Invecchiamento, Neurologiche, Ortopediche e della Testa-Collo, Fondazione Policlinico Universitario “A. Gemelli” IRCCS, 00168 Rome, Italy; andrea.bellieni@gmail.com (A.B.); beatricedicapua@gmail.com (B.D.C.); 2 Multidisciplinary Breast Center, Dipartimento Scienze della Salute della Donna, del Bambino e di Sanità Pubblica, Fondazione Policlinico Universitario “A. Gemelli” IRCCS, 00168 Rome, Italy; martin.sanchez@policlinicogemelli.it (A.M.S.); gianluca.franceschini@policlinicogemelli.it (G.F.); riccardo.masetti@policlinicogemelli.it (R.M.); 3 Istituto per la Sicurezza Sociale, 47890 Cailungo, Città di San Marino, San Marino; el.allocca@gmail.com; 4 Dipartimento di Scienze Biotecnologiche di Base, Cliniche Intensivologiche e Perioperatorie, Università Cattolica del Sacro Cuore, 00168 Rome, Italy; enrico.distasio@unicatt.it; 5 Dipartimento di Diagnostica per Immagini, Radioterapia Oncologica ed Ematologia, Fondazione Policlinico Universitario “A. Gemelli” IRCCS, 00168 Rome, Italy; fabio.marazzi@policlinicogemelli.it (F.M.); luca.tagliarri@policlinicogemelli.it (L.T.); giuseppeferdinando.colloca@policlinicogemelli.it (G.F.C.); 6 Università Cattolica del Sacro Cuore, 00168 Rome, Italy; roberto.bernabei@unicatt.it

**Keywords:** sarcopenia, physical performance, frailty, older cancer patients

## Abstract

Sarcopenia is a geriatric syndrome characterized by losses of quantity and quality of skeletal muscle, which is associated with negative outcomes in older adults and in cancer patients. Different definitions of sarcopenia have been used, with quantitative data more frequently used in oncology, while functional measures have been advocated in the geriatric literature. Little is known about the correlation between frailty status as assessed by comprehensive geriatric assessment (CGA) and sarcopenia in cancer patients. We retrospectively analyzed data from 96 older women with early breast cancer who underwent CGAs and Dual X-ray Absorptiometry (DXA) scans for muscle mass assessment before cancer treatment at a single cancer center from 2016 to 2019 to explore the correlation between frailty status as assessed by CGA and sarcopenia using different definitions. Based on the results of the CGA, 35 patients (36.5%) were defined as frail. Using DXA Appendicular Skeletal Mass (ASM) or the Skeletal Muscle Index (SMI=ASM/height^2), 41 patients were found to be sarcopenic (42.7%), with no significant difference in prevalence between frail and nonfrail subjects. Using the European Working Group on Sarcopenia in Older People (EWGSOP2) definition of sarcopenia (where both muscle function and mass are required), 58 patients were classified as “probably” sarcopenic; among these, 25 were sarcopenic and 17 “severely” sarcopenic. Only 13 patients satisfied both the requirements for being defined as sarcopenic and frail. Grade 3-4 treatment-related toxicities (according to Common Terminology Criteria for Adverse Events) were more common in sarcopenic and frail sarcopenic patients. Our data support the use of a definition of sarcopenia that includes both quantitative and functional data in order to identify frail patients who need tailored treatment.

## 1. Introduction

Breast cancer is the most frequent cancer diagnosed in women and the leading cause of cancer death among women [1]. About 60% of these new diagnoses involve patients > 65 years of age and about 40% of patients are >70 [2].

Chronological age per se is a misleading criterion when deciding the best treatment for older women with breast cancer. A group of older patients with the same cancer of identical chronologic age can demonstrate wide heterogeneity concerning vitality, comorbidity, functional status, physiologic reserve, and psychosocial functioning [3,4,5]. Nonetheless, the accrual of older adults in cancer trials has been poor and undermined by several barriers through the years [6]. This is a severe matter of concern when evidence-based guidelines are applied to older populations, with negative consequences on survival [7]. Thus, a personalized approach based on individual patients’ clinical conditions and functionality rather than age [8,9,10,11] should be considered the standard of care for older women with breast cancer.

To help guide treatment decisions, two geriatric medicine features have been incorporated in geriatric oncology: the concept of frailty and the comprehensive geriatric assessment. The Comprehensive Geriatric Assessment (CGA) represents the most efficient evaluation instrument, as recommended by the International Society of Oncological Geriatrics (SIOG) [12] and recently by the American Society of Clinical Oncology (ASCO) [13], to identify and define the frailty of the patient and his/her functional reserve [14]. Despite accumulating evidence regarding the value of the geriatric assessment in terms of encompassing older patients’ diversity, a full CGA is considered rather time-consuming. Its effectiveness is far limited without interdepartment collaborative care and frailty-targeted optimized intervention programs to implement daily oncology practices [15,16,17,18,19,20,21]. CGA is the only method capable of assessing older cancer patients’ frailty, predicting the risk of toxicity related to the treatments and the risk of mortality [22]. The CGA approach is considered essential to identify problems that are not immediately evident. Several studies have demonstrated the ability of CGA to identify otherwise unrecognized conditions of vulnerability to support the decision-making of the specialist (oncologist, radiotherapist, surgeon) when estimating the risk of toxicity to prevent said toxicity and preserve the functional performance of patients [23,24,25,26].

CGA can help to identify several geriatric syndromes [27]. Among all of them, sarcopenia has played an increasing role [28]. Sarcopenia is now considered one of the biological mechanisms underlying the concept of frailty. A reduction, compared to physiological criteria, in skeletal muscle mass characterizes this, with essential structural changes in muscle quality, and typically manifests itself with an alteration in function and/or a reduction in strength [29,30]. Several studies have shown the association between sarcopenia and functional decline, disability, frailty, falls, risk of fractures, multiple hospitalizations, and death [31,32]. A high prevalence of sarcopenia has been described in cancer patients, and its occurrence is associated with an increased risk of treatment toxicity, increased postoperative complications, increased sensitivity to antiblastic treatments, and a higher mortality rate, regardless of cancer stage [33]. It should also be stressed that cancer and cancer treatments may themselves be responsible for increasing disability, thereby accelerating the functional decline trajectory.

Several definitions of sarcopenia have been proposed. Initially, low muscle mass was considered the only criterion for diagnosis [34]. This is also the case for the vast majority of reports on cancer populations, with different indexes and cut-offs proposed. By contrast, in the geriatric field, the role of physical performance and muscle strength has been stressed as a necessary complement to the definition. The original operational definition of sarcopenia by the European Working Group on Sarcopenia in Older People (EWGSOP) [35] in 2010 was a significant change at the time, adding the muscle function to the former definitions, which were based only on detection of low muscle mass [28,29,31,32]. In its 2018 definition (Table 1), EWGSOP2 uses low muscle strength as a primary parameter of sarcopenia. It is considered a more reliable measure of muscle function and a better predictor of adverse outcomes [36,37]. Specifically, sarcopenia is probable when low muscle strength is detected. A sarcopenia diagnosis is confirmed by the presence of low muscle quantity or quality. When low muscle strength, low muscle quantity/quality, and low physical performance are all detected, sarcopenia is considered severe (Table 1). Techniques for evaluating muscle quantity are available in many but not all clinical settings. As instruments and methods for assessing muscle quality are developed and refined in the future, this parameter is expected to grow in importance as a defining feature of sarcopenia. Physical performance was formerly considered part of the core definition of sarcopenia. In the revised guidelines, it is used to categorize the severity of sarcopenia.

The present study aimed to assess sarcopenia’s prevalence using different definitions in a population of older women with breast cancer and investigate possible correlations between sarcopenia and frailty status and the impact of these conditions on toxicities from oncological treatments.

## 2. Materials and Methods

We retrospectively analyzed data on the comprehensive geriatric evaluation of older women admitted at the Breast Surgery Unit of the Fondazione Policlinico Universitario A. Gemelli IRCCS, starting in January 2016 and ending in December 2019. All breast cancer patients aged ≥ 70 with a histological confirmed early breast cancer (stage 0–III, according to TNM) underwent CGA. The patients were selected weekly during the multidisciplinary tumor board (TBM), based on the registry criteria, and sent for geriatric evaluation. The only exclusion criteria were: life expectancy less than six months and refusal to participate in the study. Anthropometric measures (weight, height, BMI), the socio-family context, and support of all the patients were recorded and investigated. The patients underwent a medical examination, including medical history and physical examination. The primary socio-demographic data, the comorbidities, and the information on the oncological history and the anatomo-pathological and cancer immunohistochemical features, in accordance with the data present in the patients’ medical records, were detected. Anthropometric measures (weight, height, body mass index) were collected for all patients. The comprehensive geriatric assessment (CGA) was based on recommendations from SIOG and national clinical guidelines [38]. The following areas were evaluated: performance status by Eastern Cooperative Oncology Group (ECOG) [39,40], comorbidity burden by the Charlson Comorbidity Index [41], functional status by Activity of Daily Living (Katz ADL) [42] and by Instrumental Activities of Daily Living (Lawton IADL) [43], cognition by Mini-Mental State Examination (MMSE) [44], nutritional status by Mini Nutritional Assessment (MNA) [45], mood by Geriatric Depression Scale (GDS) [46], physical performance by Short Physical Performance Battery (SPPB), gait speed and time up-and-go test (TUGT) [47,48,49], muscular strength by handgrip (Jamar dynamometer) [50] and chair stand test [51]. Patients were asked about the presence of common geriatric syndromes, such as falls or incontinence. Only patients who completed a Dual X-ray Absorptiometry (DXA) scan for muscle mass evaluation were included for the present study.

### 2.1. Sarcopenia and Frailty Definitions

The definition of sarcopenia by EWGSOP2 [28] was applied, using cut-offs proposed by the guidelines mentioned above (Table 1). Muscle mass was measured by (DXA) total body (Hologic Horizon) Appendicular Skeletal Mass (ASM), calculated as the sum of arm and limb lean mass measured through DXA and expressed in kg. Frailty was defined by Balducci’s criteria [52,53] considered as the detection of deficits in two or more domains of the CGA.

### 2.2. Toxicities

We retrospectively analyzed hospital electronic medical records of the patients included in the present study after a 12-month follow-up period in order to detect toxicities as they were reported by treating clinicians. Toxicities were evaluated using Common Terminology Criteria for Adverse Events (CTCAE) v5.0.

### 2.3. Analysis

All evaluations were performed by geriatricians belonging to the geriatric oncology team of the Fondazione Policlinico Universitario A. Gemelli IRCCS and who were specialized in the field of geriatric oncology and appropriately trained within the training courses of the International Society of Oncological Geriatrics (SIOG) [54]. Once the data collection was completed, all analyses were carried out using IBM SPSS 23. The collected data were synthesized using means and standard deviations for continuous variables and absolute and percentage frequencies for categorical variables. Statistical significance was conventionally set at *p* < 0.05.

## 3. Results

From January 2016 to December 2019, over 300 elderly patients aged ≥ 70 years belonging to the Breast Surgery Unit of Fondazione Policlinico Universitario A. Gemelli IRCCS (Rome), were evaluated.

Using the inclusion criteria, 96 patients were enrolled. The medium age of the examined sample was 76.9 (70 ÷ 89; SD 4.586), with an average level of comorbidity measured by the Charlson Comorbidity Index (CCI) of 6.7 (5 ÷ 13; SD—1.904), while ECOG performance status was mainly between 0 and 1 (89.6% of patients). Invasive ductal carcinoma was the most common histotype (75%), followed by lobular carcinoma (14.6%) (Table 2).

All patients underwent surgery: 84.38% (81) received a conservative treatment (quadrantectomy), representing 12.3% of cases (10 patients) with total lymphadenectomy, while 41.9% of cases (34 patients) received the removal of the sentinel lymph node. A total of 14.58% (14) received a full mastectomy, of whom three also underwent total lymphadenectomy. Less than 20% of patients received adjuvant chemotherapy, while almost two-thirds received adjuvant radiotherapy. In total, 85.4% of the patients were prescribed hormone suppressive therapy (with an aromatase inhibitor), based on hormone receptor status.

Based on CGA results, 35 patients (36.5% of the sample) were defined as frail, according to Balducci’s criteria, and 61 (63.5) as nonfrail (Table 3).

Frail patients were older compared to nonfrail ones (79 years, SD 4.994; 75.67, SD 3.88; *p* = 0.000) and had a slightly higher burden of comorbidities (mean CCI of frail patients was 7.71 against 6.11 for nonfrail patients, *p* = 0.10) and a higher level of disability (ADL mean 5 vs. 5.72 for nonfrail; IADL mean 5.4 vs. 7.64 for nonfrail; *p* = 0.000), and were at higher risk of malnutrition (MNA mean 23.12 vs. 25.87; *p* = 0.001).

The cognitive level of frail patients assessed by the MMSE screening test was almost 2 points lower than the other patients (25.09 frail patients; 27.88 nonfrail patients; *p* = 0.001) and they had a higher frequency of depressive symptoms than the nonfrail ones (average GDS 6.19 vs. 3.47 for nonfrail; *p* = 0.000). Polypharmacy, defined as taking five or more medications daily, was the case for 74.3% of frail patients and 49.2% of nonfrail patients.

Using the DXA parameters (either appendicular skeletal mass (ASM) or Skeletal Muscle Index {SMI = ASM/height^2]), 41 of 96 patients undergoing evaluation by DXA were found to be sarcopenic (42.7% of the sample examined) and 55 nonsarcopenic (57.3%). The average SMI of the sample was 6.47 (4.91 ÷ 9.73; SD 0.893). There were no significant differences in the prevalence of sarcopenia between frail and nonfrail patients (see Table 3)

**Figure 1:**
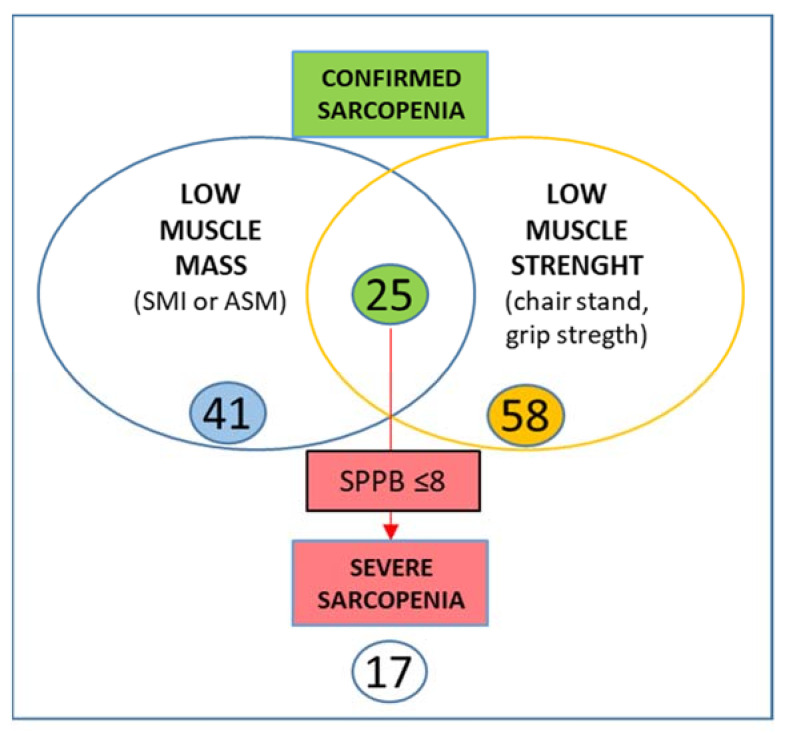
Prevalence of sarcopenia according EWGSOP2 definition [16]. SMI = =Skeletal Muscle Index; ASM = Appendicular Skeletal Muscle mass; SPPB = Short Physical Performance Battery

According to the revised EWGSOP2 [28] criteria, 58 patients could be classified as “probably” sarcopenic with low muscle strength, defined as a chair stand test > 15 s for five rises (average value 17.09 s; 15.07 ÷ 26.7; SD 4.175). Among them, only 25 (out of 58) had a confirmed diagnosis of sarcopenia (either ASM < 15 kg or SMI DXA < 5.5 kg/m^2^) with an average ASM of 13.46 kg and an average SMI value of 5.6 kg/m^2^. In total, 17 (out of 25) patients could be defined as severely sarcopenic with an SPPB score ≤ 8 (mean value 4.7) (Figure 1).

Frail sarcopenic patients had a mean ASM of 12.89 kg (SD 1.087) and a mean SMI value of 5.49 kg/m^2^ (SD 0.376). Frail nonsarcopenic patients had a mean ASM of 17.39 kg (SD 2.47) and a mean SMI value of 7.11 kg/m^2^ (SD 0.991).

**Figure 2:**
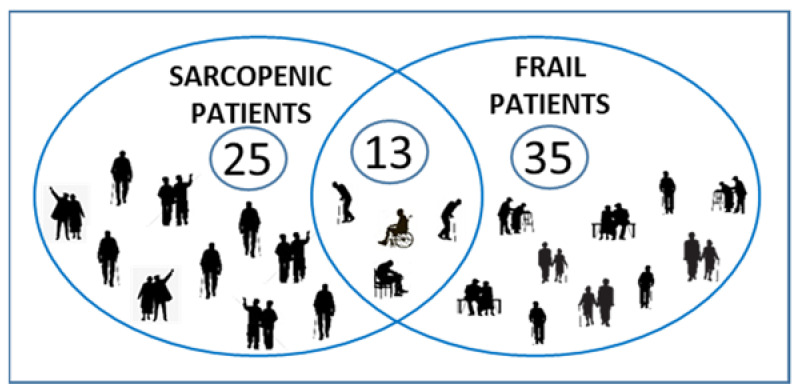
Prevalence of frailty and sarcopenia in the study sample.

Figure 2 shows the overlap between sarcopenia and frailty (as assessed by the results of CGA). Only 13 patients satisfied both the requirements for being defined sarcopenic (“confirmed” sarcopenia along to EWGSOP2) and frail (using modified Balducci’s criteria derived from CGA). Among the sarcopenic population, the proportion of patients that are also frail increases, moving from “probable” sarcopenia to “severe” sarcopenia (proportion of frail patients is 55.2% for “probable”, 56.5% for “confirmed”, and 72.2% for “severe” sarcopenia).

In a one-year follow-up, the whole sample reported 52 cases of treatment toxicities (54.16%). According to the Common Terminology Criteria for Adverse Events (CTCAE), in the frail group 17 out of 35 patients developed toxicities of any types: five patients had grade 3–4 toxicities (14%). Among sarcopenic patients, 12 out of 23 patients developed toxicities of any types; five patients experienced grade 3–4 toxicities (22%).

**Figure 3:**
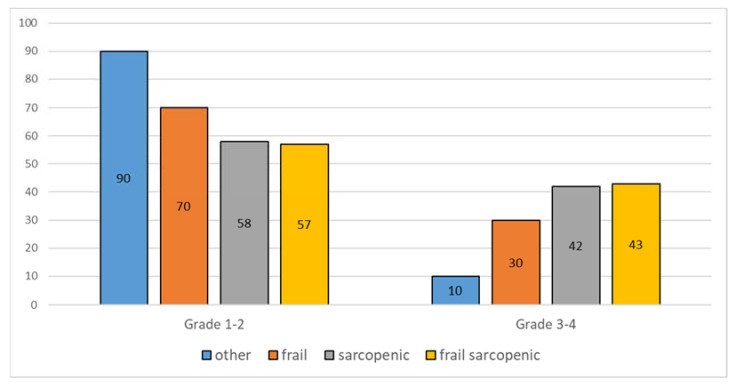
Percentage of patients who experienced Grade 1–2 and Grade 3–4 toxicities (according to Common Terminology Criteria for Adverse Events, CTCAE) in our population.

Among patients reporting toxicities, frail patients reported Grade 3–4 toxicities (according to CTCAE) more frequently than nonfrail (29% vs. 14%) ones, while sarcopenic patients reported G3–G4 toxicities more than nonsarcopenic patients (42% vs. 13%) (Figure 3).

## 4. Discussion

In the aging scenario of the general population and the increasing number of diagnosed and treated cancers in older adults, it has become critical to identify, understand, and assess the so-called geriatric syndromes. Among these, more attention is being placed on sarcopenia. For this reason, it has become essential to know the differences between sarcopenia and the loss of muscle mass related to the normal process of muscle aging or other pathological conditions such as cachexia [30].

In our sample, we identified different frequencies of sarcopenia depending on the definition used. Sarcopenia can be defined as a pathological loss of skeletal muscle mass characterized by essential structural changes in muscle quality, which occurs in older adults and shows functional impairment and/or strength reduction [55]. Aging is related to a decline in muscle mass and strength [56] but only when this decline becomes pathological (sarcopenia) does this process lead to adverse health outcomes [57].

In patients with cancer, many studies showed how the loss of muscle mass is a prevalent condition independent of disease stage and body mass [58]. This is due to many factors leading to the deterioration of muscles: inflammation, cancer-derived catabolic factors, malnutrition, reduced physical activity, and the effect of cytotoxic and targeted treatments on muscle mass and quality [10,59].

Loss of muscle mass can precede the cancer and further complicate its course, predisposing patients to a shorter time of tumor progression, increased chemotherapy-related toxicity, postoperative complications, poor functional status, hospitalization, increased length of hospital stay, high 30-day readmission rate, and mortality [60]. While the loss of muscle mass has been proven to be an independent predictor of adverse outcomes at all ages and for several cancers, such as breast cancer, hepatocellular carcinoma, and advanced urothelial cancer [60,61], the presence of sarcopenia in older cancer patients, who could be at higher risk for this condition, has been associated with therapy-related toxicities and increased adverse outcomes [33], [61,62,63].

In oncology, the skeletal muscle index (SMI) is the main parameter used to evaluate sarcopenia. Several epidemiologic studies have determined the prevalence of sarcopenia using cut-off values determined by CT scans or DXA when muscle mass is normalized for height [64,65]. Muscle function has rarely been taken into consideration. This is mainly due to most existing studies’ retrospective natures, relying on large CT scan datasets for oncological reasons (disease staging or surgical evaluation). At the same time, physical function tests are seldom conducted in routine clinical practice.

In recent years, first in the geriatric field and then in other settings, the definition of sarcopenia has shifted from an evaluation of muscle mass to a qualitative assessment of muscle function. Physical performance is a powerful predictor of adverse outcomes. This concept has been an integral part of the “physical frailty phenotype” construct [32]. Indeed, sarcopenia can be considered the most relevant biological determinant of physical frailty. Moreover, measurement of muscle mass and quality has many technological limitations, while the selection of specific cut-offs is a matter of debate [66], while muscle function is much easier to measure, at least in geriatric clinics (where hang grip or chair standing tests are routinely conducted). This new definition of sarcopenia correlates with many adverse outcomes (institutionalization risk, toxicity, mortality). This aspect has meant that sarcopenia, from a simple geriatric syndrome, has become one of the fundamental bases of modern geriatrics.

Still, in the nongeriatric setting, there is often confusion between frailty and sarcopenia, so we designed this study to try to identify those factors that can identify patients at greater risk of adverse outcomes.

Our study shows different ways to define sarcopenia and that the quantitative data (i.e., muscle mass measurement) alone is not sufficient. We detected a high prevalence of low muscle mass in the whole sample (almost 42.7%), almost equally distributed in frail and nonfrail patients. The proportion of patients with reduced muscle mass is in line with what has been reported in the literature [67]. However, when more stringent criteria that incorporate muscle function (such as EWGSOP 2) were used, only a limited proportion of patients (26%) could still be defined as “sarcopenic”. Indeed, severely sarcopenic patients were almost always classified as frail on the results of CGA.

Even though sarcopenia has been regarded as a key component of the frailty status in older adults, it should be kept in mind that frailty is a multidimensional concept that goes beyond each of its features. Relying solely on what is usually defined as “sarcopenia” in oncological research (that is, low muscle mass) can be misleading, resulting in classifying many more patients as frail and possibly omitting valuable treatments. On the contrary, a stricter definition, where muscle loss is coupled with reduced muscle function (both strength and performance), could enable a better selection of patients.

In this study, 13.5% of patients have a correspondence between sarcopenia and frailty; this group has a very high probability of experiencing adverse outcomes. Identifying this subgroup will allow a real personalization of treatments in the near future and the identification of the risk of adverse events for apparently fit patients.

DXA is a noninvasive instrument for body composition assessments. Information on muscle and adipose tissue can also be gathered by other tools such as CT scans. Identifying reduced muscle mass should be promoted in oncology to avoid the adverse outcomes associated with this condition [28]. For example, chemotherapy could be personalized based on body composition, with doses adapted to the individual patient to limit toxicities [68]. Frailty is better identified by CGA which allows the detection of unidentified problems and the correct malignancy prognosis estimation [69]. Thus, CGA avoids over- and undertreatments in a scenario focus on tailored treatments.

In our sample, both frailty and sarcopenia are associated with treatment-related toxicities, especially with more severe (G3–G4) ones. However, this association is stronger for sarcopenic or sarcopenic-frail patients than it is for frail patients, although these differences are not statistically significant. It should be kept in mind that treating clinicians were aware of frailty status, so that more potentially toxic treatments were spared to frailer patients. On the other side, it is also possible that sarcopenia was not routinely considered when planning surgery or adjuvant therapies. This could have resulted in more adverse effects both in sarcopenic nonfrail patients and in sarcopenic frail patients, which indeed showed a comparable frequency of high-grade treatment-related toxicities.

The novelty of this study is in having identified for the first time in the same group of breast cancer patients the various degrees of sarcopenia and frailty through the available gold standards and a subgroup at high risk of adverse events (toxicity, reduced compliance, etc.) between the two.

Some limitations of our study have to be acknowledged. Firstly, given the cross-sectional nature of our data, it was not possible to make inferences on otherwise clinically significant outcomes associated with frailty and sarcopenia, such as survival or loss of functional independence. Secondly, the small sample size prevents us from generalizing our results to other clinical situations. Indeed, we believe that these data should prompt further research on the association between frailty, sarcopenia, and body composition, hopefully with a longer follow patients report to identify which parameter constitutes the best clinical deterioration predictor. More research is also needed on possible interventions to counteract sarcopenia, restoring muscle mass, and function. Physical exercise is a promising intervention that could prevent functional decline in older adults [70]. More data on the effect of structured physical activity in older adults with cancer are needed.

## 5. Conclusions

Our data support the use of a comprehensive definition of sarcopenia that takes into account both physical performance and muscle mass in order to identify older women with breast cancer at higher risk of clinical deterioration and treatment-related toxicities. A multidimensional geriatric assessment in this population is strongly recommended and evaluation of muscle mass and function should be regarded as an essential part of it, with the aim of offering patients the best personalized treatment.

## Figures and Tables

**Table 1:** Definition of sarcopenia by European Working Group on Sarcopenia in Older People (EWGSOP2) guidelines ^1^.

Criteria:	Suggested Measures and Cut-offs (for Women)
(1) Low muscle strength	Grip strength, <16 kg Chair standing, >15 s for five rises
(2) Low muscle quantity or quality	ASM (appendicular skeletal muscle mass), <15 kg SMI (Skeletal Muscle Index): ASM/height^2^, <5.5 kg/m^2^
(3) Low physical performance	Gait speed, ≤0.8 m/s Short Physical Performance Battery (SPPB), ≤8 points score Timed Up-and-Go Test, ≥20 s 400 m walk test, noncompletion or ≥6 min for completion
Definitions: Probable sarcopenia is identified by Criterion 1. Confirmed sarcopenia: both Criterion 1 and Criterion 2 are satisfied. Severe sarcopenia: if Criteria 1, 2 and 3 are all met.	

^1^ Cruz-Jentoft et al., (2019). Sarcopenia: revised European consensus on definition and diagnosis. Age and Ageing, 48(1):16–31.

**Table 2:** Characteristics of the study population.

	N. of Patients	%
Histotype	96	100
Invasive Ductal Carcinoma	72	75
Invasive Lobular Carcinoma	14	14.6
other	10	9.6
STAGE		
0	1	1
I	45	46.9
II	37	38.5
IIIa	10	10.4
IIIb	3	3.1
ECOG Performance status		
0–1	86	89.6
≥2	9	9.4
Breast Surgery		
Conservative	82	85.4
Mastectomy	14	14.58
Axillary Surgery		
None	17	17.7
Sentinel Lymph Node	38	39.6
Lymph Node Sampling	21	21.9
Lymphadenectomy	13	13.5
Adjuvant Treatments		
Chemotherapy	19	19.8
Radiotherapy	62	64.6
Hormone therapy	82	85.4
Toxicities	52	100
Grade 1–2	42	81
Grade 3–4	10	19
		

ECOG: Eastern Cooperative Oncology Group.

**Table 3:** Characteristics of frail and nonfrail patients (based on comprehensive geriatric assessment (CGA) results).

Parameters		Nonfrail Patients			Frail Patients			
	N	N	Mean	Std. Dev.	N	Mean	Std. Dev.	*p* < 0.05
AGE	96	61	75.6	3.88	35	79	4.994	0.000
CCI	96	61	6.11	1.462	35	7.71	2.163	0.01
FRIED criteria	96	61	1.13	1.049	35	2.88	1.066	0.000
ADL	96	61	5.72	0.488	35	5	0.97	0.000
IADL	96	61	7.64	0.895	35	5.4	2.316	0.000
MMSE	96	61	27.88	2.345	35	25.09	3.76	0.001
MNA	93	60	25.87	2.262	33	23.12	3.517	0.001
GDS	85	58	3.47	2.617	27	6.19	3.903	0.000
SPPB	96	61	9.38	1.823	35	4.66	2.3	0.000
TUGT	75	49	10.29	2.227	26	16.76	6.018	0.000
SPEEDs	90	58	4.27	1.099	32	7.32	3.532	0.000
HANDGRIP	66	40	17.51	4.695	26	11.77	5.279	0.002
BMI	96	61	28.18	4.598	35	28.71	5.723	0.01
POLYPHARMACY Mean number of drugs	96	61	4.79	2.583	35	6.34	2.449	0.001
SMI	96	61	6.46	0.73	35	6.51	1.134	0.959
ASM	96	61	15.7	2.1	35	15.7	3	0.988

CCI = Charlson Comorbidity Index; ADL = Activities of Daily Living; IADL = Instrumental Activities of Daily Living; MMSE = Mini-Mental State Examination; MNA = Mini Nutritional Assessment; GDS = Geriatric Depression Scale; SPPB = Short Physical Performance Battery; TUGT = Time Up and Go Test; BMI = Body Mass Index; SMI = Skeletal Muscle Index; ASM = Appendicular Skeletal Muscle mass.
